# High-performance electrochemical sensing platform based on poly(arginine)@nickel ferrite nanocomposite-modified electrode for the detection of bisphenol a in food and environmental matrices

**DOI:** 10.1039/d6ra04423a

**Published:** 2026-07-06

**Authors:** Md. Romzan Ali, Md. Ruhul Amin, Kibreya Kabir Kanok, Suraiya Yasmin Setu, Tamanna Jahan Tuli, Md. Ikram Hossain, Md. Rafiul Hasan, Mohamed Aly Saad Aly, Md. Zaved H. Khan

**Affiliations:** a Laboratory of Nano-bio and Advanced Materials Engineering (NAME), Department of Chemical Engineering, Jashore University of Science and Technology Jashore 7408 Bangladesh mohamed.alysaadaly@ece.gatech.edu zaved.khan@just.edu.bd rafiul.che@just.edu.bd; b School of Electrical and Computer Engineering, Georgia Institute of Technology Atlanta GA 30332 USA; c Department of Electrical and Computer Engineering at Georgia Tech Shenzhen Institute (GTSI) Shenzhen Guangdong 518055 China

## Abstract

Bisphenol A (BPA) is a widespread industrial compound exploited mostly in the fabrication of epoxy resins and polycarbonate plastics, which are normally present in food containers, beverage bottles, and water pipes. As a renowned endocrine-disrupting chemical, BPA leaches from these materials into food and water, acting as a xenoestrogen that mimics natural estrogen in the human body, thus necessitating effective monitoring and removal strategies. Herein, a poly(arginine)@nickel ferrite, P-Arg@NiFe_2_O_4_, nanocomposite-based electrochemical sensor was constructed for identifying BPA in fruits and wastewater. The sensor leverages the magnetic and conductive properties of NiFe_2_O_4_ and the P-Arg's molecular recognition capability to enable sensitive detection. The synthesized materials were characterized using a complementary suite of analytical techniques such as XRD to analyze the crystalline structure, SEM to visualize surface morphology, and FT-IR spectroscopy to analyze the chemical composition and functional groups. A comprehensive approach to assess the performance, stability, and sensing mechanism of the developed sensor *via* pH optimization, cyclic voltammetry, and electrochemical impedance spectroscopy techniques was employed. Using differential pulse voltammetry, the sensor displayed a strong linear relationship with peak current and BPA levels across two ranges: 1.0 × 10^−3^ to 1.0 × 10^−1^ µM (*R*^2^ = 0.99962) and 5 × 10^−1^ to 1 × 10^2^ µM (*R*^2^ = 0.98757), with a 3.84 × 10^−4^ µM detection limit. Recovery trials using fruit and wastewater samples verified the sensor's practical usability. Moreover, when numerous interfering substances and metal ions were present, the sensor demonstrated an outstanding selective detection of BPA. This is the first report on the fabrication and subsequent application of a P-Arg@NiFe_2_O_4_ nanocomposite for electrochemical identification of BPA.

## Abstract 2

Bisphenol A (BPA) is a widespread industrial compound exploited mostly in the fabrication of epoxy resins and polycarbonate plastics, which are normally present in food containers, beverage bottles, and water pipes.

## Graphical abstract

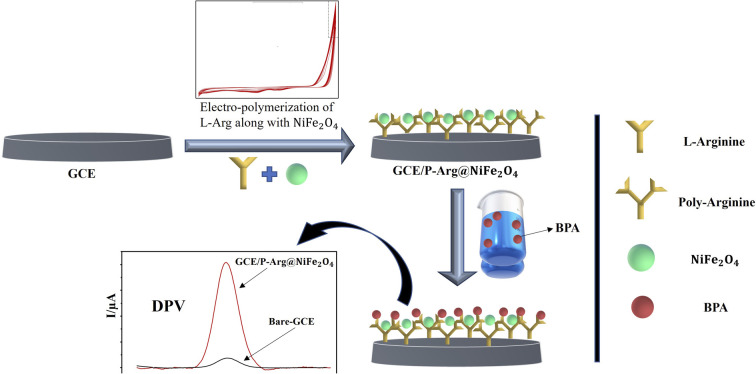

## 1. Introduction

Bisphenol A (BPA), or 4,4'-(propane-2,2-diyl) diphenol, is a synthetic industrial compound, produced *via* the condensation of phenol and acetone. Due to its chemical structure, BPA can leach into food and beverages, particularly when heated.^1^ Human exposure to bisphenol A (BPA) is extensive, with the primary route being dietary intake, followed by non-dietary environmental exposure.^2^ BPA acts as a potent endocrine-disrupting chemical (EDC), mimicking the hormone estrogen and interfering with natural hormonal processes. The endocrine-disrupting effects of BPA are linked to numerous health issues, including heart problems, metabolic disorders like diabetes, reproductive issues, and neurodevelopmental problems, with particular concern for developmental exposure during fetal and childhood stages.^3^ BPA is primarily used to fabricate polycarbonate plastics and epoxy resins found in a collection of commercial products, for example thermal paper, water bottles, food packaging, CDs, and medical equipment.^4,5^ The pervasive presence of BPA has led to significant contamination of aquatic ecosystems, including sediment and diverse aquatic organisms. An estimated 8 million tons of BPA are synthesized globally each year.^6^ In response to the potential health hazards of BPA, the European Food Safety Authority (EFSA) established in 2015, a 4.0 µL kg^−1^ body weight of a tolerated daily intake (TDI).^7^ Given its widespread use and possible toxic effects, there is an urgent need for reliable, economical, adaptable, and rapid screening tools that can detect traces of this endocrine disruptor in food, water, and environmental samples.

Numerous methods, for instance high-performance liquid chromatography,^8^ immunoassays,^9^ gas chromatography–mass spectrometry,^10^ and fluorescence-based methods,^11^ were established for the determination of BPA. Although these approaches are renowned for their selectivity and sensitivity, their applicability for routine analysis is limited because they frequently call for expensive equipment, complex sample preparation techniques, qualified staff, and large time investments.^12–14^ Recently, there have been an increased interest in immunoassay-based detection techniques; however, their performance may be hampered by antibody instability and cross-reactivity with similar compounds, resulting in decreased specificity and reliability.^15,16^

Electrochemical sensing has arisen as a superior substitute to conventional techniques or detecting BPA due to their ability to provide rapid, sensitive, and on-site analysis along with low detection limits, ease of use, and flexibility in sensor design.^17–19^ These characteristics make electrochemical sensors ideal for the use in biomedical diagnostics and environmental monitoring.^20–23^ Over the past 20 years, electrochemical sensing has undergone a significant transformation, driven by the integration of nanomaterials that enhance sensor sensitivity, selectivity, and response times. The last 20 years have witnessed a transformative evolution in electrochemical sensing, driven by the integration of advanced nanomaterials designed to enhance sensor performance, lower detection limits, and increase sensitivity. The systematic alteration of the morphology (size, shape) and surface properties of metals, metal oxides, conductive polymers, carbon-based nanostructures, and metal–organic frameworks (MOFs) allows for tailored interactions with target analytes, overcoming limitations of traditional electrode materials.^24^ The development of electrochemical sensor technologies has been significantly accelerated by the increasing query for point-of-care diagnostics, real-time monitoring, single-molecule detection, wearable devices.^25^

Recently, polymerized amino acids have emerged as powerful tools in electrochemical sensing, providing biodegradable, non-toxic, and biocompatible platforms. Various polymers such as 2-hydroxyethylmethacrylate,^26^l-cystine,^27^ pyrrole,^28^ and reduced graphene oxide^29^ have been utilized for the detection of BPA, mostly in molecularly imprinted polymer (MIP)-based sensors. However, MIP sensors are frequently hindered by incomplete template extraction, poor analytical reliability, sluggish binding kinetics, and structural instability. Additionally, MIP sensors utilizing basic PEDOT-modified electrodes are heavily constrained by a high detection limit of 55 µM,^30^ which renders them inadequate for trace-level analytical applications. Consequently, current electrochemical sensors struggle to achieve high sensitivity, selectivity, and stability simultaneously without relying on complex template-based designs. In contrast, poly(l-arginine)-modified electrodes deliver exceptional electrocatalysis, sensitivity, and stability,^31,32^ achieving a limit of detection (LOD) of 3.84 × 10^−4^ µM. While arginine provides high biocompatibility and stabilizes nanostructures through electrostatic interactions and carboxylate repulsion,,^33^ the bare poly(l-arginine) polymer suffers from limited electrocatalytic surface area. To enhance polymer-modified electrodes for the detection of BPA, researchers have used CuFe_2_O_4_,^34^ Au/TiO_2_ nanotubes,^35^ Au nanoparticles,^27^ and carbon materials^34^_._ However, these suffer from critical limitations, such as CuFe_2_O_4_ leaches toxic metal ions in acidic buffers, Au/TiO_2_ nanotubes require costly, multi-step synthesis with poor electron transfer; Au nanoparticles need additional modifiers and aggregate easily, carbon materials are hydrophobic and poorly dispersible. In contrast, spinel ferrites offer high chemical stability, surface reactivity, scalability, and cost-effectiveness.^36–41^ Among these, the inverse spinel structure of NiFe_2_O_4_ (Ni^2+^ at octahedral sites, Fe^3+^ at tetrahedral/octahedral sites) is exceptional, providing strong magnetic permeability, high electrical resistivity, thermal stability, low coercivity, corrosion resistance, and minimal eddy current loss.^42,43^ As an n-type semiconductor, it excels in sensing, catalysis, and biomedical applications.

The innovative aspect of the present work is associated with the novel use of poly(arginine) conjugated with NiFe_2_O_4_ for BPA determination. While the advantageous properties of nickel ferrite NiFe_2_O_4_, such as high surface area, rapid electron transfer, magnetism, chemical stability, and cost-effectiveness, have been extensively investigated, its integration with poly(arginine) remains unexplored. This specific polymer functionalization leverages selective guanidinium–BPA interactions, thereby overcoming the critical limitations of toxicity, high cost, poor selectivity, and instability inherent in previously reported materials. This study aims to develop a highly sensitive and cost-effective electrochemical sensing platform by modifying a glassy carbon electrode (GCE) with a novel P-Arg@NiFe_2_O_4_ material. The performance of the modified GCE was evaluated using cyclic voltammetry (CV) and electrochemical impedance spectroscopy (EIS). The developed electrochemical sensor showed high sensitivity with a broad linearity range, and a very low detection limit of 3.84 × 10^−4^ µM. Moreover, the electrochemical sensor provided a strong resistance to interferences such as Ca^2+^, Na^+^, Cu^2+^, K^+^, Fe^2+^, Mg^2+^, Al^3+^, Zn^2+^, ascorbic acid, and uric acid, in addition to high stability during 15 consecutive days, compared with other sensors like the MIP-based and PEDOT-based sensors.

## 2. Material and methodology

### 2.1. Materials

Chemicals utilized in the investigation was lab grade and did not require any additional purification. l-Arginine was obtained from Aladdin Reagents (Shanghai, China), and bisphenol A (BPA) came from Guanru Chemical (Shanghai, China). Phosphate buffer solution (PBS) was bought from Sigma-Aldrich, China. Merck (India) supplied additional common reagents, like potassium chloride and sodium hydroxide. For all electrochemical studies, 0.1 M PBS, prepared with ultrapure water at pH 7.4 level, was used.

### 2.2. Apparatus

The electrochemical measurements were conducted employing triple-electrode equipped Corrtest CS300 electrochemical workstation (Hubei, China). Glassy carbon (2.00 mm, type CS920), platinum wire, and Ag/AgCl served as the working, counter, and reference electrodes, correspondingly. The facially functionalized electrodes were morphologically analyzed utilizing GeminiSEM 500 scanning electron microscope (SEM, ZEISS, Oberkochen, Germany), ZAHNER impedance analyzer EIM6ex (Kronach, Germany) was utilized to examine electrochemical investigations conducted by the impedance spectroscopy (EIS). A Bruker D8 Advanced System was used for X-Ray Diffraction (XRD) analysis, while a Thermo Nicolet iS50 FTIR spectrometer was used for Fourier Transform Infrared Spectroscopy (FT-IR) analysis.

### 2.3. Nickel ferrite nanoparticles synthesis

**Scheme 1:**
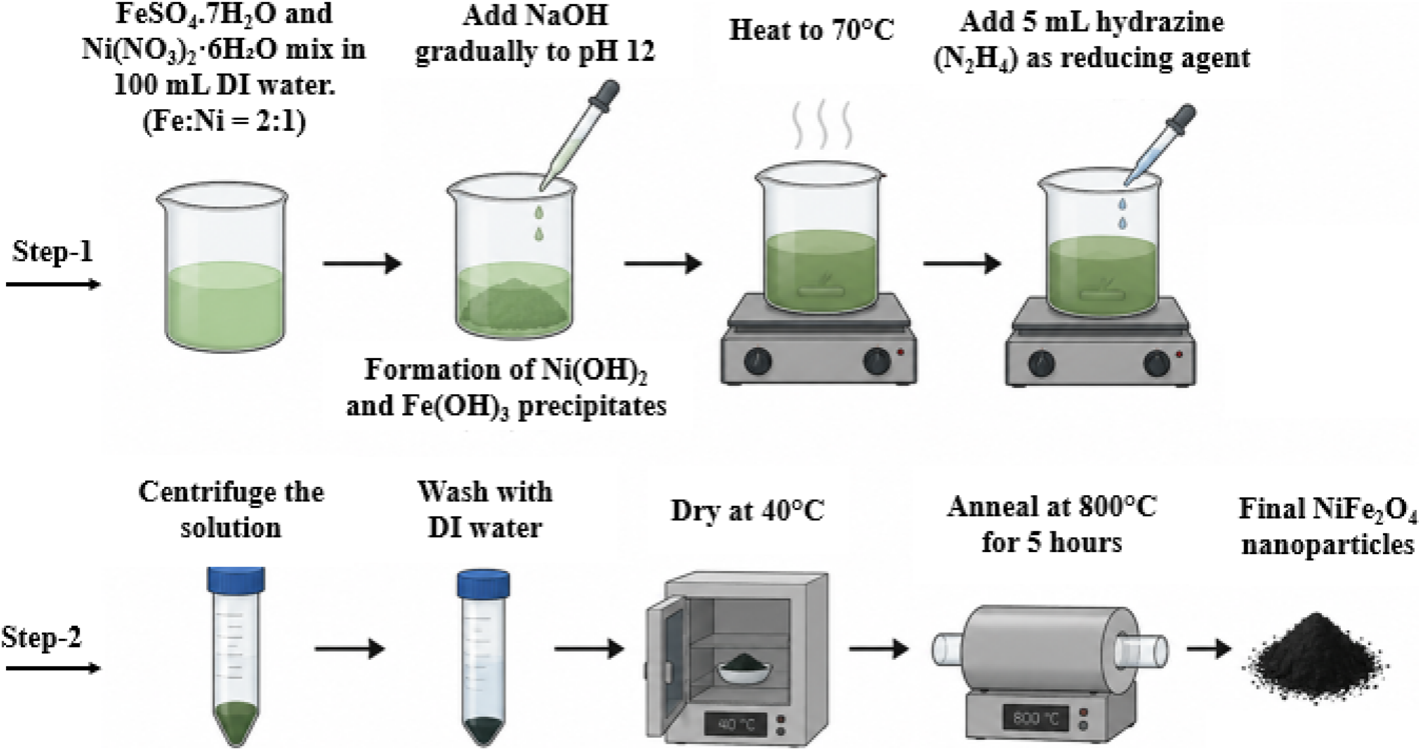
Synthesis of nickel ferrite (NiFe_2_O_4_) nanoparticles.

Based on a previously published procedure with minor variations,^44^ NiFe_2_O_4_ nanoparticles were synthesized using a co-precipitation technique. The starting solution contained ferrous sulfate heptahydrate (FeSO_4_·7H_2_O) and nickel nitrate hexahydrate (Ni(NO_3_)_2_·6H_2_O) in a 2 : 1 molar ratio (Fe : Ni), dissolved in 100 mL of distilled water. In the co-precipitation step, nickel and iron hydroxides (Ni(OH)_2_ and Fe(OH)_3_) were formed through gradual addition of sodium hydroxide to a pH of 12, resulting in a complete precipitation of metal ions from the aqueous solution. The solution was heated up to 70 °C and then mixed with 5 mL of hydrazine (N_2_H_4_) as a reduction agent in order to prevent the oxidation of Fe^2+^ and to avoid the occurrence of nuclei. After centrifugation, washing, and drying at 40 °C, the samples were annealed at 800 °C for 5 hours in oxygen atmosphere. The annealing temperature was chosen based on thermal analysis indicating complete conversion to cubic spinel NiFe_2_O_4_ at temperatures exceeding 750 °C. Furthermore, an annealing duration of 5 hours was determined to be optimal for producing highly crystalline samples with minimal particle growth. The synthesis process is schematically illustrated in Scheme 1.

### 2.4. Sensor fabrication

Prior to the electrode modification, a micro cloth was employed to sequentially polish the surface of the pristine GCE with 10^3^, 300, and 50 nm alumina powders suspended in water. After rinsing the electrode with ultrapure water, it underwent ultrasonic cleaning for 3 minutes in ethanol and DI water. Following air drying, the clean GCE was electrochemically activated by cycling for 23 cycles in 0.01 M H_2_SO_4_ with 0.1 V s^−1^ of scan rate by utilizing CV in a voltage window of −1.0–1.0 V *vs.* the voltage of the reference electrode. To fabricate the sensor, the activated GE was submerged in 0.1 M (pH 7.4) PBS that included 4.3 × 10^5^ µM of prepared NiFe_2_O_4_ nanoparticles and 5 × 10^3^ µM of l-arginine. In a voltage window of −2.0–2.0 V (*vs.* the potential of the reference electrode), the electrodeposition was carried out for 12 cycles *via* CV at a rate of scan of 0.1 V s^−1^.

### 2.5. Electrochemical measurements

For electrochemical study, the facially functionalized electrodes were submerged in 0.1 M PBS with varying concentrations of Bisphenol A. Sensor responsiveness was evaluated at 25 °C temperature in a nitrogen atmosphere using differential pulse voltammetry (DPV) at a 100 mv/s of scan rate and within a voltage window of −0.2–0.6 V.

## 3. Findings and discussion

### 3.1. Characterization of synthesized NiFe_2_O_4_ and P-Arg@NiFe_2_O_4_/GCE

**Fig. 1:**
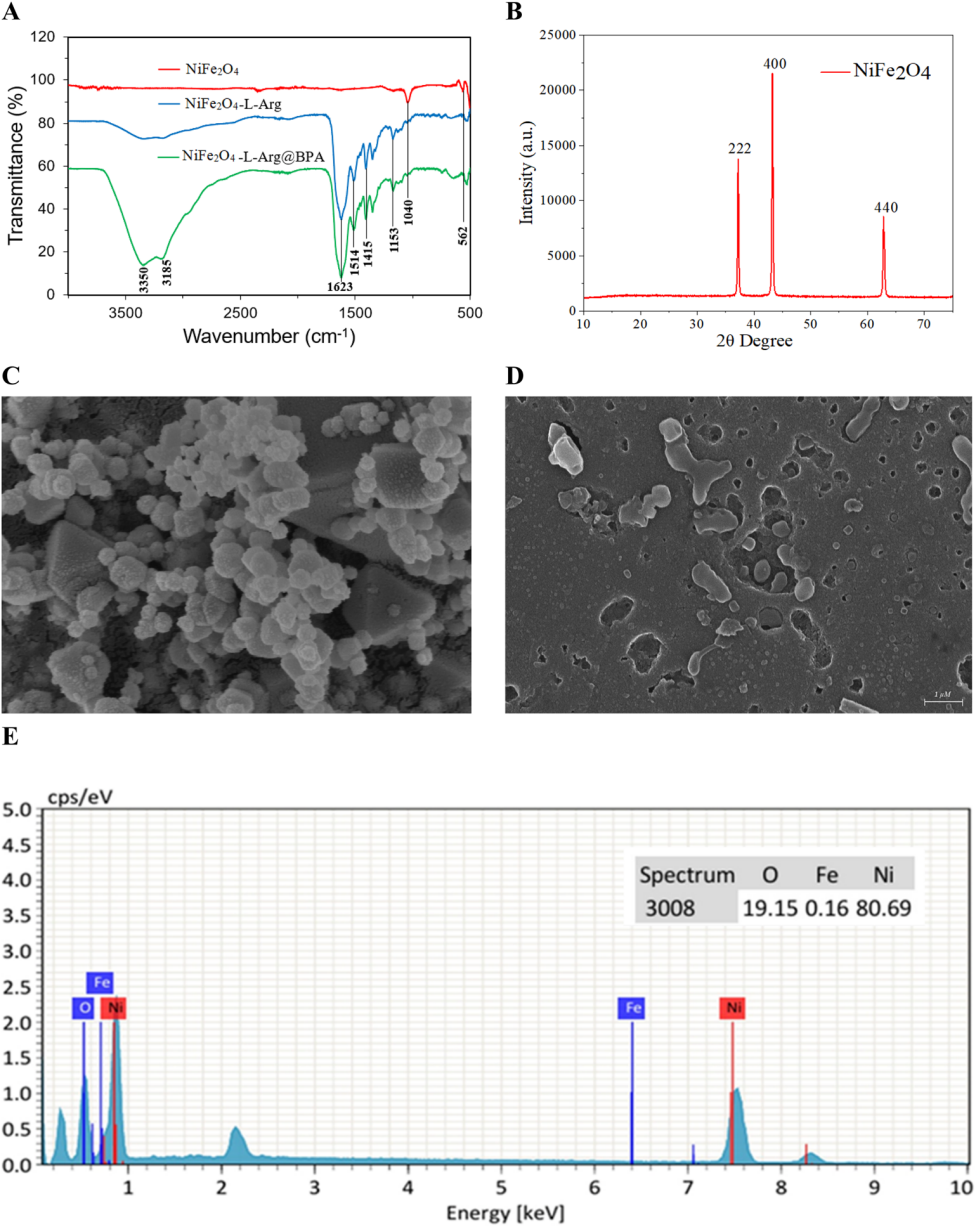
FT-IR spectra of (A) (i) NiFe_2_O_4_, (ii) NiFe_2_O_4_@L-Arg; (iii), and NiFe_2_O_4_@L-Arg/BPA; XRD spectrum of (B) NiFe_2_O_4_; SEM of (C) NiFe_2_O_4_; (D) P-Arg@NiFe_2_O_4_/GCE; and EDX analysis of (E) NiFe_2_O_4_.

Fourier-transform infrared (FTIR) spectroscopy was performed to confirm the successful surface modification of the nickel ferrite nanoparticles with l-arginine and to investigate the subsequent adsorption mechanism of Bisphenol A (BPA). Fig. 1(A) illustrates the comparative FTIR spectra of pristine NiFe_2_O_4_, l-arginine-functionalized nickel ferrite L-Arg@NiFe_2_O_4_, and ferrite L-Arg@NiFe_2_O_4_-BPA. The spectrum of the pristine NiFe_2_O_4_ (red curve) displays a relatively flat profile with high transmittance, featuring a prominent characteristic absorption band at 562 cm^−1^. This sharp band is assigned to the intrinsic metal–oxygen M-O stretching vibrations at the tetrahedral sites of the spinel ferrite lattice, confirming the formation of the crystalline oxide core.^45^ Following the functionalization with l-arginine ferrite (L-Arg@NiFe_2_O_4_, blue curve), several distinct organic absorption bands emerge, verifying successful surface modification. A broad band appearing in the 3200–3500 cm^−1^ region is attributed to the overlapping stretching vibrations of hydroxyl –OH groups and primary amines –NH_2_ from the amino acid backbone.^46^ The sharp, highly intense peak at 1623 cm^−1^ corresponds to the asymmetric stretching vibration of the carboxylate groups COO-or the bending deformation modes of the amino/guanidino groups. Furthermore, the peaks at 1415 cm^−1^ and 1153 cm^−1^ are assigned to the symmetric COO– stretching and C–N stretching vibrations, respectively, validating the anchoring of l-arginine onto the ferrite nanoparticle surface.^47^ Upon the addition of BPA (L-Arg@NiFe_2_O_4_-BPA, green curve), a significant drop in overall transmittance along with a sharp enhancement of existing vibrational modes is observed, indicating BPA conjugated with L-Arg@NiFe_2_O_4_. The high-wavenumber region resolves into two distinct, intense bands at 3350 cm^−1^ and 3185 cm^−1^, which are assigned to the stretching vibrations of phenolic –OH groups from BPA involved in hydrogen bonding networks with the l-arginine moiety.^48^ The unshift peaks in the spectra collectively confirm that BPA was successfully captured by the L-Arg@NiFe_2_O_4_ surface.

The X-ray diffraction pattern for the synthesized NiFe_2_O_4_ nanoparticles is shown in Fig. 1(B). Three sharp diffraction peaks were detected around 2*θ* positions of 37.22°, 43.25°, and 62.82° that belonged to the (222), (400), and (440) crystal planes, respectively^49,50^ according to JCPDS (Card No. 10-0325). The crystal size calculated using Debye–Scherrer equation and Cu Kα radiation with *λ* equal to 1.5406 Å, were found to be 19.5 nm, 17.4 nm, and 25.2 nm, respectively, as summarized in Table 1. The high intensity peak around 2*θ* of 43.1° confirms that this crystal plane has the highest diffraction orientation. From the sharp peaks, it is clear that a well-crystalline structure is achieved. Moreover, since there is no other crystal planes detected, there are no other secondary crystals formed.

**Table 1:** Crystallographic parameters obtained from XRD analysis

Crystal plane (*hkl*)	Diffraction angle 2*θ* (degree)	FWHM (degree)	Crystal size, *D* (nm) (approximately)
(222)	**37.22**	0.43	19.5
(400)	**43.22**	0.49	17.4
(420)	**62.82**	0.37	25.2

Surface morphology of the synthesized NiFe_2_O_4_ and the P-Arg@NiFe_2_O_4_/GCE electrode was assessed by utilizing SEM. The NiFe_2_O_4_ surface is depicted in Fig. 1(C), where crystalline nanoparticles with distinct grain boundaries and haphazard aggregation are visible. In certain areas, these particles' magnetic characteristics result in dense and loosely bound agglomerates.^51^ Although the majority of the nanoparticles were spherical, some cylindrical or elliptical shapes were also noted. Additionally, the SEM images show a uniform distribution of nanoparticles with distinct boundaries between each particle. For electrocatalytic applications, the synthesis produced a desirable structure with a large surface area that resembles sheet-like formations scattered over a wide area.^52^Fig. 1(D) shows the P-Arg@NiFe_2_O_4_/GCE electrode after the l-arginine layer is electrochemically deposited. The SEM picture shows a regular, net-like interconnected structure that confirms good polymer layer deposition on the electrode, in line with earlier findings.^53^ Following NiFe_2_O_4_ deposition, the surface roughness increased, indicating effective film formation.

The composition and cleanliness of the nanoparticles were clarified by Energy-Dispersive X-ray Spectroscopy (EDX) conducted at room-temperature, as shown Fig. 1(E). Lack of pollutants was confirmed by the EDX spectra, which only showed peaks related to Ni, Fe, and O.^54^ Consequently, the production of the modified P-Arg@NiFe_2_O_4_/GCE electrode and the synthesis of NiFe_2_O_4_ were effectively verified.

### 3.2. Fabrication of P-Arg@NiFe_2_O_4_/GCE sensor

**Fig. 2:**
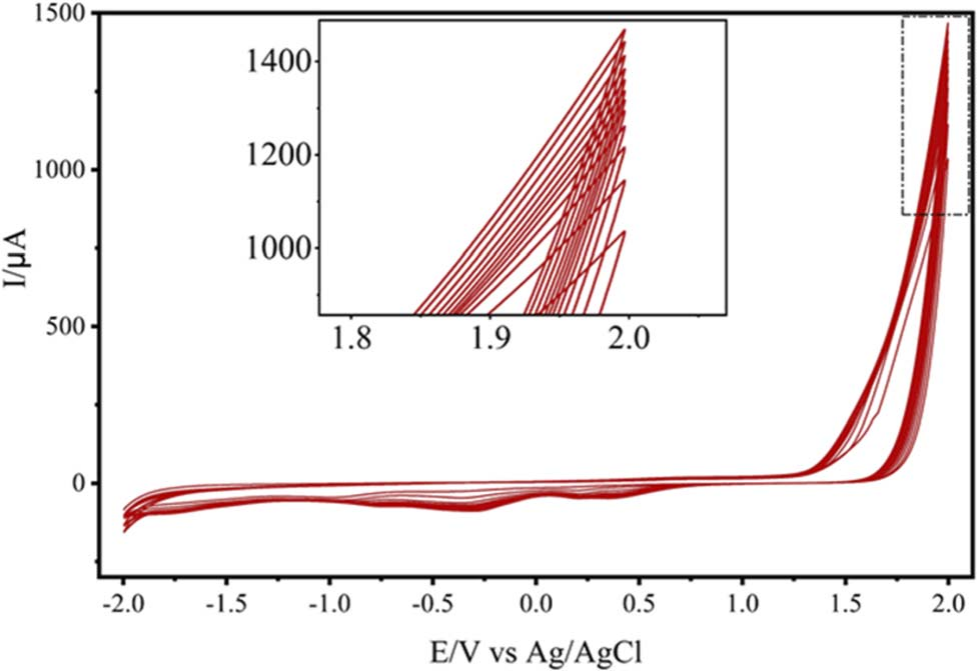
Electrochemical polymerization's cyclic voltammograms of a mixture that contains 4.3 × 10^5^ µM NiFe_2_O_4_ and 5 × 10^3^ µM l-arginine on GCE.

As seen in Fig. 2, l-arginine was electropolymerized on the surface of the pristine GCE using CV by scanning for 12 cycles from −2.0 to +2.0 V at 0.1 V s^−1^ in a pH 7.4 solution of 0.1 M PBS containing 4.3 × 10^5^ µM NiFe_2_O_4_ and 5 × 10^3^ µM L-Arg. The initial cathodic and anodic peaks at −0.48 and +1.52 V (*vs.* the references electrode voltage), respectively, produced by forming a P-Arg layer on the surface of the pristine GCE. The growth of the cathodic and the anodic peaks in the CV curves demonstrated the progressive synthesis of P-Arg on GCE surface. With every scan, the peak current rose, suggesting the formation of an electroactive polymer sheet. A conductive polymer layer was confirmed when the oxidation peak stabilized after it was entirely created. NH_2_ groups electro-polymerized to generate covalent C–N bonds with the surface of the electrode as part of the polymerization development.^55^ It is well established in electrochemical studies that the L-Arg, monomer, undergoes oxidative polymerization at high anodic potentials, generating α-amino acid free radical, which propels polymerization. The created P-Arg coating are covalently bonded to the electrode surface.^56^ P-Arg may be electrochemically deposited onto GCE at higher potentials because it has a positively charged guanidino group. In order to accomplish effective deposition, the potential window in this study was established between −2.0 V and +2.0 V.

### 3.3. Electrochemical characterization of P-Arg@NiFe_2_O_4_/GCE

**Fig. 3:**
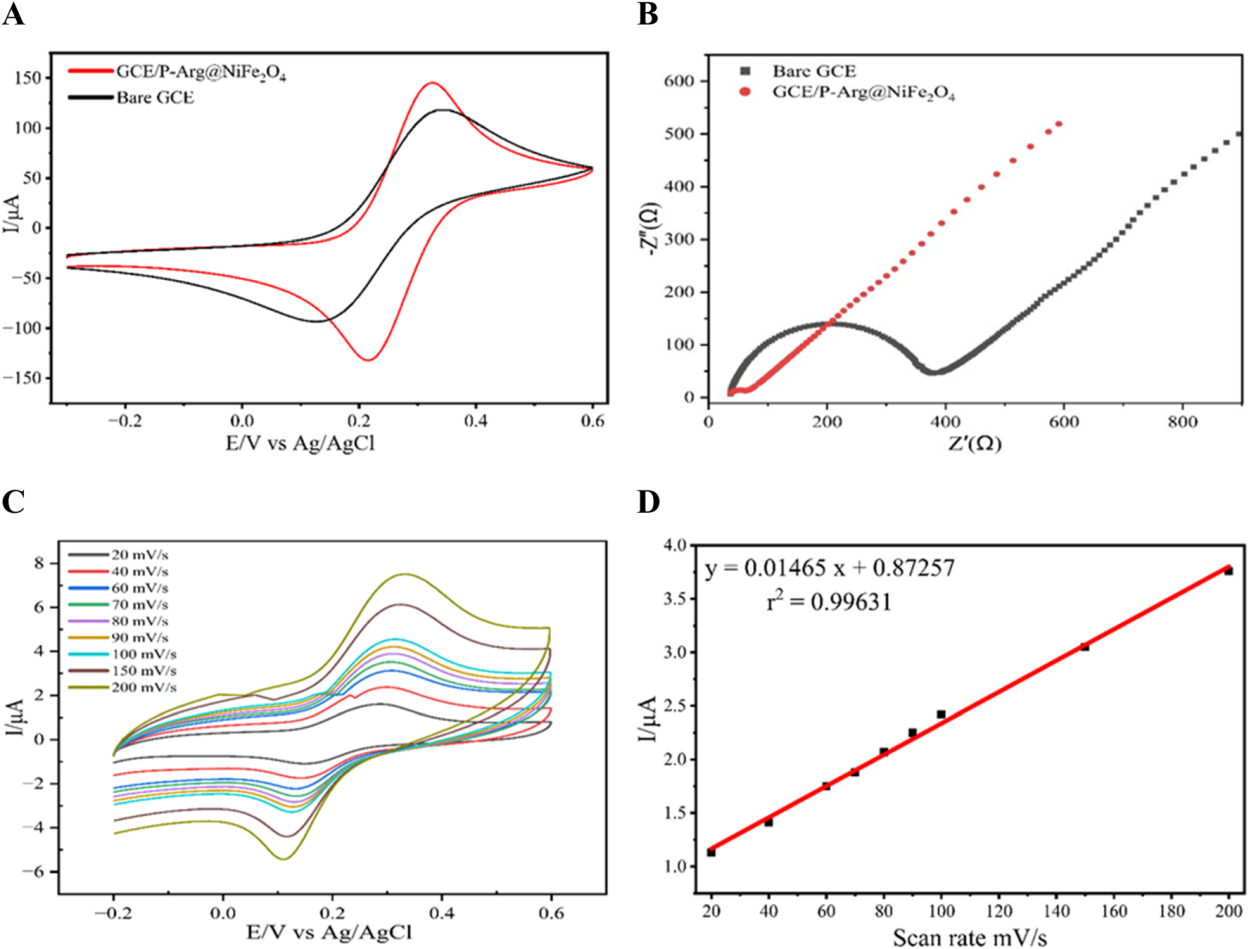
CV of (A) pristine GCE, and P-Arg@NiFe_2_O_4_/GCE modified electrode measured in 100 mmol L^−1^ KCl including 5.0 mmol L^−1^ Fe(CN)_6_^3−/4−^. Nyquist plot of EIS of (B) pristine GCE, and P-Arg@NiFe_2_O_4_/GCE modified electrode measured in 100 mmol L^−1^ KCl including 5.0 mmol L^−1^ Fe(CN)_6_^3−/4−^. Cyclic voltammetry for different scan rate at 20, 40, 60, 70, 80, 90, 100, 150 and 200 mV s^−1^ in 100 mmol L^−1^ KCl containing 5.0 mmol L^−1^ Fe(CN)_6_^3−/4−^ solution in (C) P-Arg@NiFe_2_O_4_/GCE, and (D) the equivalent calibration plot.

The electrochemical properties of both pristine and facially functionalized electrodes were investigated using CV and EIS in 5.0 mM [Fe(CN)_6_]^3−/1−^ and 0.1 M KCl solution. Every modified electrode showed clear, reversible redox peaks in Fig. 3(A). The pristine GCE showed two redox peaks with the lowest peak currents, which corresponded to [Fe(CN)_6_]^3−^/^*Ψ*−^.

The measured peak-to-peak separation (Δ*E*_p_) of the bare GCE was 219 mV, indicating slow electron transport at its surface. Interestingly, when L-Arg and NiFe_2_O_4_ were electrodeposited, a substantial raise in the redox peak currents was noted and the Δ*E*_p_ dropped to 110 mV, indicating improved electron transfer kinetics.^57^ This is because the P-Arg matrix's positive charges encourage the [Fe(CN)_6_]^3−^/Ψ^−^ electrochemical process, and the addition of NiFe_2_O_4_ increases the electroactive surface area *via* spatial confinement.^58,59^ Additionally, the Randles–Sevcik formula, shown in eqn (1), was employed to find the electrochemical active surface area (ECSA) of both the pristine and facially functionalized electrodes.^60^1
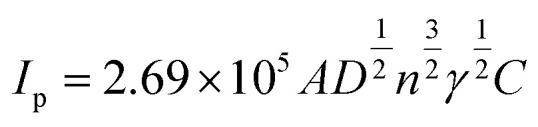
where, the electroactive surface area is denoted by *A*, the concentration of the probe molecule is presented by *C*, *D* depicts the coefficient of diffusion (6.70 ± 0.02 × 10^−6^ cm^2^ s^−1^), the scan rate is denoted by *γ* (V s^−1^), and the count of transferred electrons during redox reaction is presented by *n*. The computed *A* values for the P-Arg@NiFe_2_O_4_/GCE modified electrode and the pristine GCE were found to be 0.140 and 0.091 cm^2^, correspondingly. This result demonstrated larger ECSA and therefore improved electron transfer kinetics of the P-Arg@NiFe_2_O_4_/GCE facially functionalized electrode.

The synergistic behavior of the modified electrodes was assessed using EIS, with the corresponding results represented in Fig. 3(B). In theory, the redox probe's electron-transfer kinetics at the electrode contact are governed by the charge transfer resistance (*R*_ct_), which is shown in the Nyquist plot at higher frequencies by the semicircle's circumference. Bare GCE exhibited the largest semicircular feature, corresponding to a high *R*_ct_ value of 370 Ω, indicative of slow electron transfer. In contrast, the GCE modified with P-Arg@NiFe_2_O_4_ displayed a substantially lower *R*_ct_ of 45 Ω, highlighting the enhanced conductivity arising from the combined effect of P-Arg and NiFe_2_O_4_, which significantly facilitates electron transfer.^61^

The modified P-Arg@NiFe_2_O_4_/GCE electrode in a pH 7.4 PBS including 1.0 × 10^5^ µM BPA was employed to inspect the impact of the rate of scan on the oxidation of BPA. Fig. 3(C) shows the CV response at a scan rate ranged from 20 to 200 mV s^−1^, with current increasing proportionally to the scan rate. As scan rates continue to rise, both the anodic and cathodic peak currents showed a linear and proportionate increase. Concurrently, the values of anodic peak potentials corresponding to BPA exhibited a noticeable turn toward more positive, whereas the cathodic peak potentials moved modestly toward more negative values. This collective behavior is characteristic of a quasi-reversible electrochemical process, reflecting the influence of kinetic limitations on the redox system.^62,63^ The connection between the peak current (*I*_p_) and square root of the scan rate (*Δ*^1/2^) was methodically investigated in order to determine whether diffusion or adsorption controls the electrochemical response at the facially functionalized electrode.

A clear linear correlation was observed over the rate of scan ranged from 0.02 to 0.2 V s^−1^, as illustrated in Fig. 3(D). This linear dependence, expressed quantitatively by eqn (2), suggesting that the electrochemical process occurring at the P-Arg@NiFe_2_O_4_/GCE facially functionalized electrode is controlled by adsorption phenomena rather than diffusion.^64^

2 *I*_p_ (µA) = 0.01465 (mV s^−1^) + 0.87257

In order to examine the effect of accumulation time on the electrochemical behavior of BPA, a 10 µM concentration of BPA was used. In the experiment, the accumulation time was varied in the range of 0–150 seconds to determine the effect of accumulation time on the oxidation peak current. The oxidation peak current significantly depended on the accumulation time, as seen in Table 2. Initially, the oxidation peak current dropped from 1.50 µA at 0 second to 1.22 µA at 50 seconds. However, there was a substantial increase in the oxidation peak current at 78 seconds, when the maximum current (*i.e.* 3.08 µA) was reached. It can be concluded from these results that the molecule was efficiently accumulated on the electrode surface. When the accumulation time exceeded 78 seconds, the oxidation current started to drop from 1.73 µA at 100 seconds, but then there was an increase in current up to 2.40 µA at 150 seconds; however, these values were lower than those recorded at 78 seconds. Hence, 78 seconds was chosen as the optimal time of accumulation for BPA.

**Table 2:** The impact of accumulation duration on BPA's detection

Accumulation time (s)	Current (µA)
0	1.5
25	1.33
50	1.22
78	3.08
100	1.73
125	1.99
150	2.40

### 3.4. Electrochemical detection of BPA

**Fig. 4:**
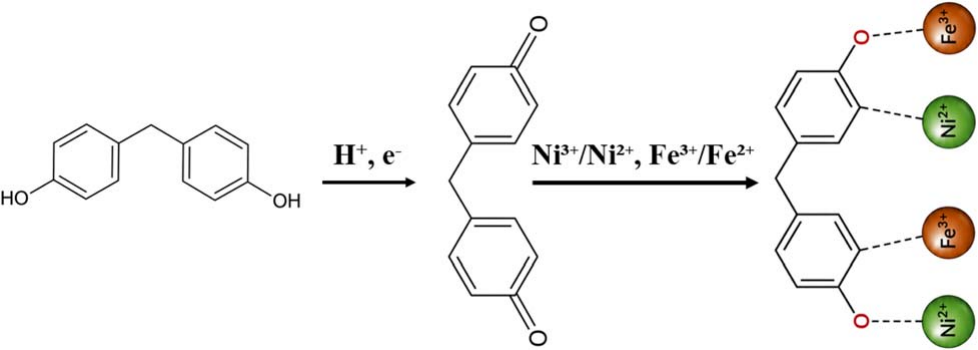
The possible reaction mechanism of BPA and NiFe_2_O_4_.

The developed P-Arg@NiFe_2_O_4_ modified electrode drives the BPA electrooxidation through sequential, single-electron transfer steps following substrate adsorption. The NiFe_2_O_4_ spinel ferrite enhanced this process by providing a large electroactive surface area. Additionally, its highly reversible Ni^3+^/Ni^2+^ and Fe^3+^/Fe^2+^ oxidation/reduction redox couples significantly accelerate electron transfer. In the electrolyte solution (BPA in 0.01 M PBS, pH 7.4), the guanidino side-chains of poly(arginine) were positively charged. This positive charge attracts the negatively charged, partially ionized BPA molecules that exist predominantly in their phenolate form. Thus, the local concentration of BPA was considerably increased at the electrode–electrolyte interface. The adsorbed BPA gives off one electron for the formation of a phenoxy radical-cation. The stabilization of this transient state is facilitated due to the resonance interaction and hydrogen bonding between phenoxy radical and guanidino groups. Subsequent electron transfer promotes phenoxy radical-cation dissociation to generate a phenoxenium cation. This cation reacts with water or hydroxide ions from the supporting electrolyte to form a dehydroxylated BPA complex. Further oxidation, releasing two electrons and two protons, then produces BPA-quinone. The possible reaction mechanism is presented in Fig. 4.^65^

**Fig. 5:**
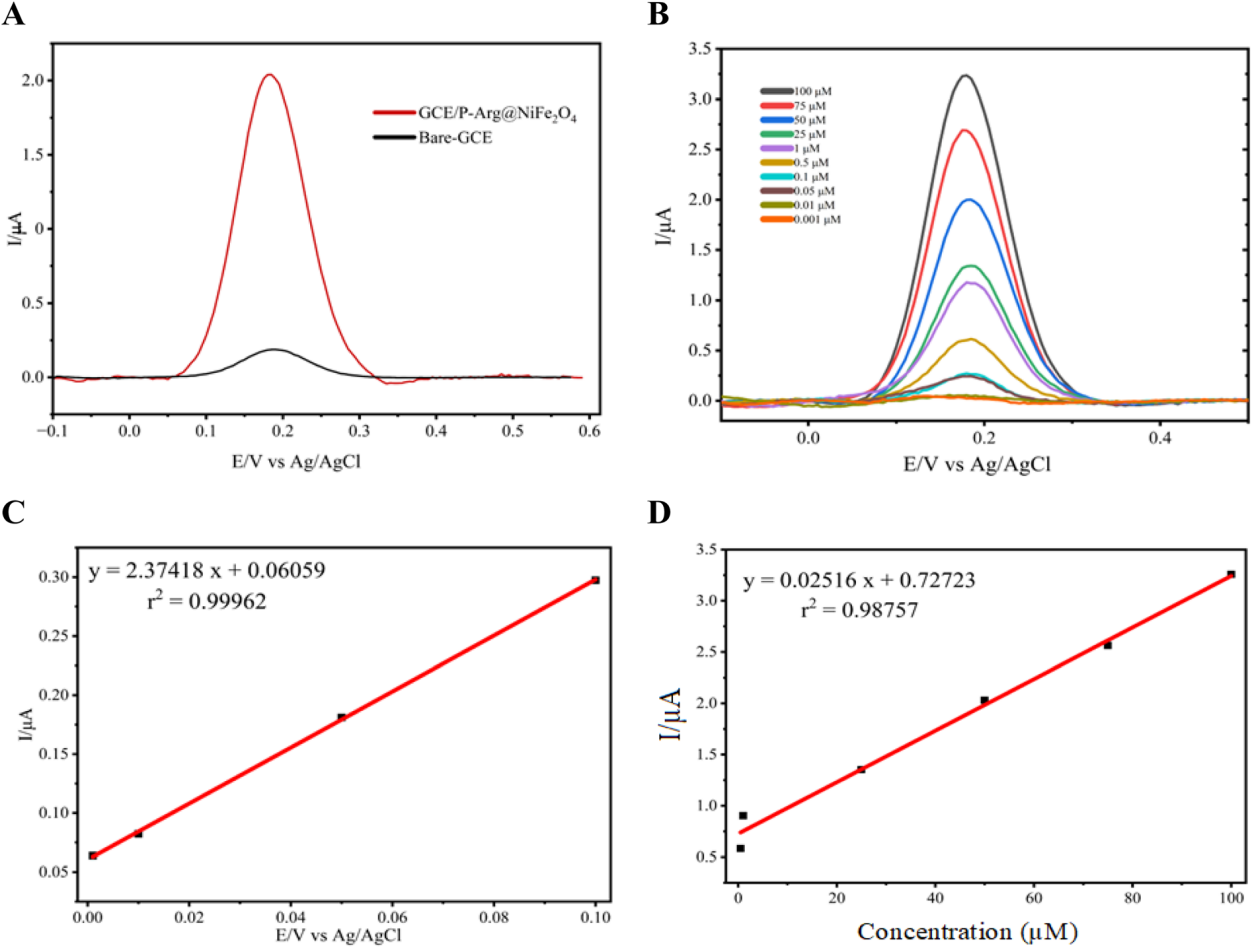
(A) DPVs of 1 mmol L^−1^ BPA at pristine GCE, and P-Arg@NiFe_2_O_4_/GCE in PBS (pH 7.4) at 0.1 V s^−1^ of scan rate; (B) DPVs for the identification of BPA by the P-Arg@NiFe_2_O_4_/GCE sensor in PBS (pH 7.4) at 0.1 V s^−1^ of a scan rate with concentration window of 1.0 × 10^−3^ to 1.0 × 10^2^ µM. (C and D) Calibration plots at different concentrations.

Using DPV and the P-Arg@NiFe_2_O_4_/GCE electrode, the electrochemical detection of BPA was methodically investigated. Different concentrations of BPA were correlated with the oxidation peak currents to create calibration plots. As depicted in Fig. 5(A), P-Arg@NiFe_2_O_4_/GCE electrode showed notably enhanced peak current in contrast to pristine GCE. The peak current at P-Arg@NiFe_2_O_4_/GCE electrode exhibited a clear linear increase as the concentration of BPA rose as shown in Fig. 5(B). Notably, the calibration plots Fig. 5(C) and (D) revealed two separate linear dynamic ranges, demonstrating the electrode's sensitivity across different BPA concentration intervals. Two linear ranges were displayed by the suggested sensor: 1.0 × 10^−3^ to 1.0 × 10^−1^ µM (*R*^2^ = 0.99962 and *I* (µA) = 2.37418 µM + 0.06059) and 5 × 10^−1^ to 1 × 10^2^ µM (*R*^2^ = 0.98757 and *I* (µA) = 0.02516 µM + 0.72723). The LOD was found to be 3.84 × 10^−4^ µM by using the equation, LOD = 3*S*/*m*; where *S* denotes the standard deviation derived from 10 consecutive blank observations, and *m* denotes the calibration curve slope.^66^

Numerous electrodes have been synthesized and utilized for the electroanalytical sensing of the target compound, as illustrated in Table 3. Graphene functionalized GCE^67^ has been described in early research studies, having a small linear range of 5.0 × 10^−2^ to 1.0 µM and a relatively large detection limit of 46.89 nM. MWCNTs@melamine/GCE^68^ improved the linear range reaching 40.0 µM, and 5.0 nM of detection limit. CuPc@MWCNT/PGE-based sensor,^69^ had a very small linear range of 2.75 µM, along with a moderate detection limit of 18.90 nM. Sensors based on r-GO/CE^70^ and WS_2_–Fe_3_O_4_–rGO/LabSPE^71^ provided moderate linear ranges but with relatively high detection limits of 8.0 nM and 30.0 nM, respectively, which make them not good fit for ultra-trace detection. Molecularly imprinted polymer-based sensors such as MIPN/NIPN/rGO^29^ and BPA@MIPPy^28^ possess lower micromolar linear ranges but a relatively higher detection limit of 8.0 nM and 29.0 nM, respectively. The current newly developed P-Arg@NiFe_2_O_4_/GCE sensor outperforms all of the aforementioned sensors. The present sensor possesses extremely large linear dynamic of 1.0 × 10^−3^ µM to 1.0 × 10^2^ µM with up to 4 orders of magnitude of enhancement, and an ultra-low detection limit of 0.38 nM. Comparing with all other systems, the present sensor has either larger dynamic range, lower detection limit, or both. Thus, the developed P-Arg@NiFe_2_O_4_/GCE electrode exhibits an excellent analytical performance in terms of wide dynamic range and low detection limit.

**Table 3:** Analytical characteristics of a few electrochemical sensors in comparison to the P-Arg@NiFe_2_O_4_/GCE sensor for the detection of BPA

Electrode material	Technique	Linear range (µM)	Detection limit (nM)	Ref.
Graphene/GCE	CV	5.0 × 10^−2^ to 1.0	46.89	67
MWCNTs @melamine/GCE	DPV	1.0 × 10^−2^ to 40.0	5.0	68
CuPc@MWCNT/PGE	DPV	2.75	18.90	69
r-GO/CE	DPV	6.0 × 10^−3^ to 4.0 × 10^−1^	8.0	70
WS_2_–Fe_3_O_4_-rGO/LabSPE	DPV	5.0 × 10^−2^ to 50.0	30.0	71
MIPN/NIPN/rGO	MIP	2.0 × 10^−2^ to 1.0	8.0	29
BPA@MIPPy	MIP	1.0 × 10^−1^ to 5.0	29.0	28
**P-Arg@NiFe_2_O_4_/GCE**	**DPV**	**1.0 × 10^−3^ to 1.0 × 10^2^**	**0.38**	**This work**

a Abbreviation: MWCNT: multi-walled carbon nanotube, CuPc: copper(ii) phthalocyanine, PGE: pencil graphite electrode, r-GO: reduced graphene oxide, CE: carbon electrode, WS: tungsten disulfide, LabSPE: laboratory screen-printed electrode, MIPN: molecularly imprinted polymer nanoparticle, NIPN: non-imprinted polymer nanoparticle, MIPPy: molecularly imprinted polypyrrole.

### 3.5. Real sample analysis

To validate the sensor in real matrices, samples of tea, pond water, river water, and juice were collected. After centrifugation (50 minutes at 6000 rpm), the supernatant was used for analysis. GCE electrodes were immersed in a 100 µM solution, then 200 µL of PBS was replaced with 100 µL of sample supernatant and 10 mM BPA. l-Arginine and nickel ferrite solutions were deposited afterward. DPV was employed to record the electrochemical response. Recovery studies involved spiking samples with BPA at three concentrations within the linear range. The analytical procedure followed that used for standards. Recovery rates ranged from 88% to 96%, confirming the sensor's applicability in complex real samples. Table 4 summarizes BPA detection results, showing satisfactory recoveries.

**Table 4:** The detection of bisphenol A in juice, river water and pond water

Sample	Added (µM)	Found (µM)	Recovery (%)
Juice	10	9.6	96
River water	10	8.8	88
Pond water	10	8.8	88

### 3.6. Reproducibility, selectivity and stability of P-Arg@NiFe_2_O_4_/GCE

Reproducibility, selectivity, and stability are critical factors for assessing the performance of facially functionalized electrode. The same electrode was used for a number of repeated DPV measurements to examine the sensor's repeatability. Five consecutive current responses of 10 µmolar BPA solution was carried out through final modified electrode and the RSD value of these five data were calculated. For the concentration sample, there was no variation in the current response (<1%).

**Fig. 6:**
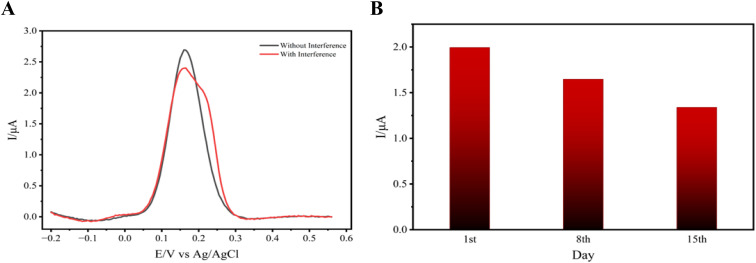
CV of (A) BPA with (i) interference of Al^3+^, Na^+^, Ca^2+^, Cu^2+^, Mg^2+^, Zn^2+^, K^+^, Fe^2+^, uric acid, and ascorbic acid; and (ii) without any interference. (B) The DPV response of P-Arg@NiFe_2_O_4_/GCE in a 10^−2^ mmol L^−1^ BPA solution measured over a 15 day period.

To assess the selectivity of the developed sensing platform, a variety of interfering substances and metal ions, including Al^3+^, Na^+^, Ca^2+^, Cu^2+^, Mg^2+^, Zn^2+^, K^+^, Fe^2+^, uric acid, and ascorbic acid were present along with BPA during DPV measurements. The electrochemical sensor's selectivity was defined by its ability to identify the target analyte (BPA) specifically when other interferents are present. An insignificant change in the BPA oxidation signal of less than 8.5%, when potential interferences were present, implying that the created sensing platform maintains high stability and accuracy, as shown in Fig. 6(A). After being refrigerated at 4 °C, the stability of the constructed sensing platform was assessed every week by making a record of the DPV response of a 1 × 10^−1^ mol L^−1^ BPA solution. The peak reaction varied by less than 15% and 25% for the first and second seven days, respectively, across a 15 day period with measurements made every seven days, as presented in Fig. 6(B). These findings show that the suggested sensor for BPA detection retains good stability, selectivity, and repeatability.

## 4. Conclusion

As a xenoestrogen, bisphenol A mimics natural estrogen by binding to estrogen receptors which can activate, overstimulate, or block hormonal pathways, leading to adverse developmental and reproductive effects, thus, it becomes a necessity to develop effective monitoring and removal strategies of BPA. As a result, in this work, a P-Arg@NiFe_2_O_4_/GCE modified electrode-based novel electrochemical sensing platform was effectively created for the selective identification of BPA even when potential interfering substances were present. The P-Arg@NiFe_2_O_4_/GCE modified electrode exhibited linear correlation between BPA concentration and current response across two ranges: 1.0 × 10^−3^ to 1.0 × 10^−1^ µM (*R*^2^ = 0.99962) and 5 × 10^−1^ to 1 × 10^2^ µM (*R*^2^ = 0.98757). Notably, the present sensor achieved a low detection limit of 3.84 × 10^−4^ µM, achieving the smallest detection limit of BPA ever reported according to the studies summarized in Table 3. Furthermore, the sensor provides a cost-efficient solution with superior long-term stability, selectivity, and repeatability. Importantly, its reliable performance in real sample analysis, yielding high recovery rates for the target analyte. When there are numerous interfering substances present, such as Ca^2+^, Na^+^, Cu^2+^, K^+^, Fe^2+^, Mg^2+^, Al^3+^, Zn^2+^, ascorbic acid, and uric acid, the developed electrochemical sensing platform demonstrated outstanding selectivity. This is the first work to report the fabrication and the subsequent application of P-Arg@NiFe_2_O_4_ nanocomposite for the electrochemical identification of BPA.

## Author contributions

Md. Romzan Ali: nanoparticles synthesis, sensor fabrication, formal analysis, analysis and discussion, and writing original draft, Md. Ruhul Amin: formal analysis, original draft editing, writing, and reviewing, Kibreya Kabir Kanok: methodology, formal analysis, writing original draft and editing, Suraiya Yasmin Setu: formal analysis, methodology, and investigation, Tamanna Jahan Tuli: investigation, and formal analysis; Md. Ikram Hossain: Data curation, and writing original draft; Md. Rafiul Hasan: supervision, project management, review, and editing; Mohamed Aly Saad Aly: conceptualization, methodology, analysis and discussion, supervision, project management, and reviewing and editing; Md. Zaved H. Khan: conceptualization, methodology, project management, supervision, review, and editing.

## Conflicts of interest

The authors have no competing interests to disclose.

## Data Availability

Upon reasonable request, the corresponding author will provide the datasets used and analyzed in this study.
